# Risk Stratification by Self-Measured Home Blood Pressure across Categories of Conventional Blood Pressure: A Participant-Level Meta-Analysis

**DOI:** 10.1371/journal.pmed.1001591

**Published:** 2014-01-21

**Authors:** Kei Asayama, Lutgarde Thijs, Jana Brguljan-Hitij, Teemu J. Niiranen, Atsushi Hozawa, José Boggia, Lucas S. Aparicio, Azusa Hara, Jouni K. Johansson, Takayoshi Ohkubo, Christophe Tzourio, George S. Stergiou, Edgardo Sandoya, Ichiro Tsuji, Antti M. Jula, Yutaka Imai, Jan A. Staessen, for the International Database of Home Blood Pressure in Relation to Cardiovascular Outcome (IDHOCO) investigators

**Affiliations:** 1 Studies Coordinating Centre, Research Unit Hypertension and Cardiovascular Epidemiology, KU Leuven Department of Cardiovascular Sciences, University of Leuven, Leuven, Belgium; 2 Tohoku University Graduate School of Pharmaceutical Sciences, Sendai, Japan; 3 Division of Hypertension, Department of Internal Medicine, University Medical Centre Ljubljana, Ljubljana, Slovenia; 4 Population Studies Unit, Department of Chronic Disease Prevention, National Institute for Health and Welfare, Turku, Finland; 5 Department of Medicine, Turku University Hospital, Turku, Finland; 6 Department of Preventive Medicine and Epidemiology, Tohoku Medical Megabank Organization, Tohoku University, Sendai, Japan; 7 Centro de Nefrología and Departamento de Fisiopatología, Hospital de Clínicas, Universidad de la República, Montevideo, Uruguay; 8 Sección Hipertensión Arterial, Servicio de Clínica Médica, Hospital Italiano de Buenos Aires, Buenos Aires, Argentina; 9 Department of Hygiene and Public Health, Teikyo University School of Medicine, Tokyo, Japan; 10 Inserm U708, University Bordeaux Segalen, Bordeaux, France; 11 Hypertension Center, Third Department of Medicine, University of Athens, Sotiria Hospital, Athens, Greece; 12 Asociación Española Primera de Socorros Mutuos, Montevideo, Uruguay; 13 Department of Public Health, Tohoku University Graduate School of Medicine, Sendai, Japan; 14 Department of Epidemiology, Maastricht University, Maastricht, The Netherlands; Bart's and The London School of Medicine and Dentistry, United Kingdom

## Abstract

**Background:**

The Global Burden of Diseases Study 2010 reported that hypertension is worldwide the leading risk factor for cardiovascular disease, causing 9.4 million deaths annually. We examined to what extent self-measurement of home blood pressure (HBP) refines risk stratification across increasing categories of conventional blood pressure (CBP).

**Methods and Findings:**

This meta-analysis included 5,008 individuals randomly recruited from five populations (56.6% women; mean age, 57.1 y). All were not treated with antihypertensive drugs. In multivariable analyses, hazard ratios (HRs) associated with 10-mm Hg increases in systolic HBP were computed across CBP categories, using the following systolic/diastolic CBP thresholds (in mm Hg): optimal, <120/<80; normal, 120–129/80–84; high-normal, 130–139/85–89; mild hypertension, 140–159/90–99; and severe hypertension, ≥160/≥100.

Over 8.3 y, 522 participants died, and 414, 225, and 194 had cardiovascular, cardiac, and cerebrovascular events, respectively. In participants with optimal or normal CBP, HRs for a composite cardiovascular end point associated with a 10-mm Hg higher systolic HBP were 1.28 (1.01–1.62) and 1.22 (1.00–1.49), respectively. At high-normal CBP and in mild hypertension, the HRs were 1.24 (1.03–1.49) and 1.20 (1.06–1.37), respectively, for all cardiovascular events and 1.33 (1.07–1.65) and 1.30 (1.09–1.56), respectively, for stroke. In severe hypertension, the HRs were not significant (*p*≥0.20). Among people with optimal, normal, and high-normal CBP, 67 (5.0%), 187 (18.4%), and 315 (30.3%), respectively, had masked hypertension (HBP≥130 mm Hg systolic or ≥85 mm Hg diastolic). Compared to true optimal CBP, masked hypertension was associated with a 2.3-fold (1.5–3.5) higher cardiovascular risk. A limitation was few data from low- and middle-income countries.

**Conclusions:**

HBP substantially refines risk stratification at CBP levels assumed to carry no or only mildly increased risk, in particular in the presence of masked hypertension. Randomized trials could help determine the best use of CBP vs. HBP in guiding BP management. Our study identified a novel indication for HBP, which, in view of its low cost and the increased availability of electronic communication, might be globally applicable, even in remote areas or in low-resource settings.

*Please see later in the article for the Editors' Summary*

## Abstract 2

Jan Staessen and colleagues compare the risk of cardiovascular, cardiac, or cerebrovascular events in patients with elevated office blood pressure vs. self-measured home blood pressure.

*Please see later in the article for the Editors' Summary*

## Editors' Summary

**Background:**

Globally, hypertension (high blood pressure) is the leading risk factor for cardiovascular disease and is responsible for 9.4 million deaths annually from heart attacks, stroke, and other cardiovascular diseases. Hypertension, which rarely has any symptoms, is diagnosed by measuring blood pressure, the force that blood circulating in the body exerts on the inside of large blood vessels. Blood pressure is highest when the heart is pumping out blood (systolic blood pressure) and lowest when the heart is refilling (diastolic blood pressure). European guidelines define optimal blood pressure as a systolic blood pressure of less than 120 millimeters of mercury (mm Hg) and a diastolic blood pressure of less than 80 mm Hg (a blood pressure of less than 120/80 mm Hg). Normal blood pressure, high-normal blood pressure, and mild hypertension are defined as blood pressures in the ranges 120–129/80–84 mm Hg, 130–139/85–89 mm Hg, and 140–159/90–99 mm Hg, respectively. A blood pressure of more than 160 mm Hg systolic or 100 mm Hg diastolic indicates severe hypertension. Many factors affect blood pressure; overweight people and individuals who eat salty or fatty food are at high risk of developing hypertension. Lifestyle changes and/or antihypertensive drugs can be used to control hypertension.

**Why Was This Study Done?:**

The current guidelines for the diagnosis and management of hypertension recommend risk stratification based on conventionally measured blood pressure (CBP, the average of two consecutive measurements made at a clinic). However, self-measured home blood pressure (HBP) more accurately predicts outcomes because multiple HBP readings are taken and because HBP measurement avoids the “white-coat effect”—some individuals have a raised blood pressure in a clinical setting but not at home. Could risk stratification across increasing categories of CBP be refined through the use of self-measured HBP, particularly at CBP levels assumed to be associated with no or only mildly increased risk? Here, the researchers undertake a participant-level meta-analysis (a study that uses statistical approaches to pool results from individual participants in several independent studies) to answer this question.

**What Did the Researchers Do and Find?:**

The researchers included 5,008 individuals recruited from five populations and enrolled in the International Database of Home Blood Pressure in Relation to Cardiovascular Outcome (IDHOCO) in their meta-analysis. CBP readings were available for all the participants, who measured their HBP using an oscillometric device (an electronic device for measuring blood pressure). The researchers used information on fatal and nonfatal cardiovascular, cardiac, and cerebrovascular (stroke) events to calculate the hazard ratios (HRs, indicators of increased risk) associated with a 10-mm Hg increase in systolic HBP across standard CBP categories. In participants with optimal CBP, an increase in systolic HBP of 10-mm Hg increased the risk of any cardiovascular event by nearly 30% (an HR of 1.28). Similar HRs were associated with a 10-mm Hg increase in systolic HBP for all cardiovascular events among people with normal and high-normal CBP and with mild hypertension, but for people with severe hypertension, systolic HBP did not significantly add to the prediction of any end point. Among people with optimal, normal, and high-normal CBP, 5%, 18.4%, and 30.4%, respectively, had a HBP of 130/85 or higher (“masked hypertension,” a higher blood pressure in daily life than in a clinical setting). Finally, compared to individuals with optimal CBP without masked hypertension, individuals with masked hypertension had more than double the risk of cardiovascular disease.

**What Do These Findings Mean?:**

These findings indicate that HBP measurements, particularly in individuals with masked hypertension, refine risk stratification at CBP levels assumed to be associated with no or mildly elevated risk of cardiovascular disease. That is, HBP measurements can improve the prediction of cardiovascular complications or death among individuals with optimal, normal, and high-normal CBP but not among individuals with severe hypertension. Clinical trials are needed to test whether the identification and treatment of masked hypertension leads to a reduction of cardiovascular complications and is cost-effective compared to the current standard of care, which does not include HBP measurements and does not treat people with normal or high-normal CBP. Until then, these findings provide support for including HBP monitoring in primary prevention strategies for cardiovascular disease among individuals at risk for masked hypertension (for example, people with diabetes), and for carrying out HBP monitoring in people with a normal CBP but unexplained signs of hypertensive target organ damage.

**Additional Information:**

Please access these websites via the online version of this summary at http://dx.doi.org/10.1371/journal.pmed.1001591.

- This study is further discussed in a *PLOS Medicine* Perspective by Mark Caulfield
- The US National Heart, Lung, and Blood Institute has patient information about high blood pressure (in English and Spanish) and a guide to lowering high blood pressure that includes personal stories
- The American Heart Association provides information on high blood pressure and on cardiovascular diseases (in several languages); it also provides personal stories about dealing with high blood pressure
- The UK National Health Service Choices website provides detailed information for patients about hypertension (including a personal story) and about cardiovascular disease
- The World Health Organization provides information on cardiovascular disease and controlling blood pressure; its A Global Brief on Hypertension was published on World Health Day 2013
- The UK charity Blood Pressure UK provides information about white-coat hypertension and about home blood pressure monitoring
- MedlinePlus provides links to further information about high blood pressure, heart disease, and stroke (in English and Spanish)

## Introduction

Current guidelines for the diagnosis and management of hypertension recommend risk stratification based on conventionally measured blood pressure, i.e., blood pressure measured in a medical environment [1],[2]. European guidelines [1] categorize blood pressure as optimal, normal, high normal, and grades 1 to 3 of hypertension; US guidelines [2] classify blood pressure as normal, prehypertension, and stages 1 and 2 of hypertension (Table 1). Blood pressure self-measured at home is a more accurate prognosticator than conventionally measured blood pressure, because of the greater number of readings and the avoidance of the white-coat effect, as well as avoidance of measurement error through use of automated blood pressure monitors [3],[4]. Affordable and validated automated monitors for blood pressure self-measurement are readily available.

**Table 1:** Classification of conventional blood pressure according to European and American guidelines and the current study.

Systolic BP (mm Hg)	Diastolic BP (mm Hg)	Blood Pressure Categories		
		European Guideline [1]	American Guideline [2]	Current Study
<120	<80	Optimal	Normal	Optimal
120–129	80–84	Normal	Prehypertension	Normal
130–139	85–89	High normal	Prehypertension	High-normal
140–159	90–99	Grade 1 hypertension	Stage 1 hypertension	Mild hypertension
160–179	100–109	Grade 2 hypertension	Stage 2 hypertension	Severe hypertension
≥180	≥110	Grade 3 hypertension	Stage 2 hypertension	Severe hypertension

BP indicates blood pressure.

The Global Burden of Diseases Study 2010 reported that high blood pressure is the leading risk factor for global disease burden, and is estimated to cause 9.4 million deaths every year—more than half of the estimated 17 million deaths a year caused by total cardiovascular disease [5]. To succeed in reducing the burden of hypertension [5],[6], efforts should be targeted where they are needed most [7]. In line with this statement [7], we examined to what extent blood pressure self-monitoring succeeds in refining risk stratification within established categories of conventional blood pressure (CBP), in particular at levels assumed to be associated with no or only mildly increased risk [1],[2]. We addressed the issue in an individual-participant meta-analysis of 5,008 people not being treated for hypertension randomly recruited from five populations and enrolled in the International Database of Home Blood Pressure in Relation to Cardiovascular Outcome (IDHOCO) [8],[9].

## Methods

### Ethics Statement

All studies included in IDHOCO received ethical approval. They have been described in detail in peer-reviewed publications. All participants gave informed written consent.

### Search Strategy and Study Inclusion Criteria

**Figure 1:**
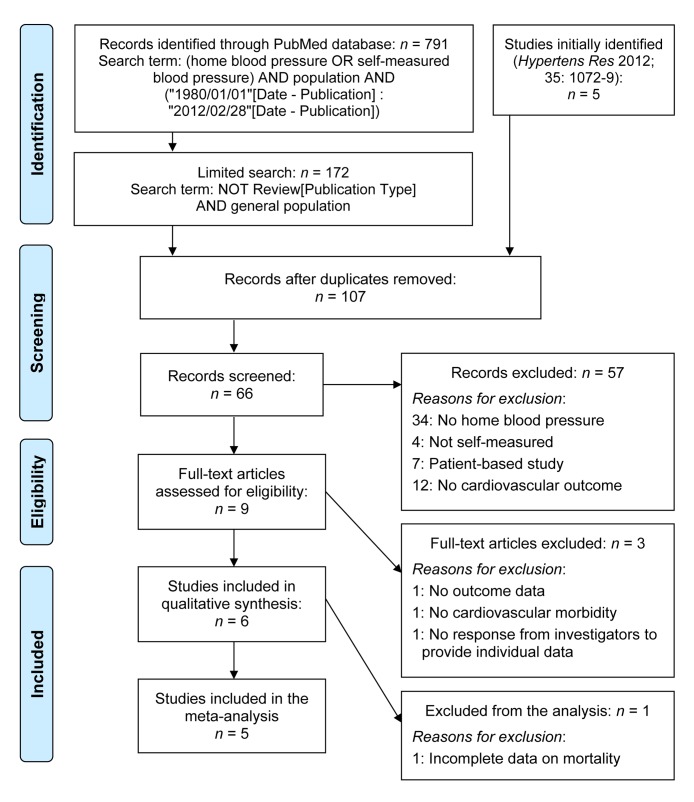
Flow diagram of selected studies and participants. Electronic searches of the literature were performed in February 2012 before publication of the IDHOCO protocol [8] and were repeated in July 2013.

Figure 1 describes the selection of studies and participants based on electronic searches of the literature done in February 2012 before publication of the IDHOCO protocol [8] and repeated in July 2013. We searched the PubMed database, using as initial search terms (home blood pressure OR self-measured blood pressure) AND population AND (“1980/01/01”[Date - Publication] : “2012/02/28”[Date - Publication]), yielding 791 publications. We then limited the search as follows: NOT review[Publication Type] AND general population, resulting in 172 hits. Two authors (K. A. and T. J. N.) independently reviewed titles and abstracts. Studies were eligible for inclusion if they met the following criteria: (1) baseline information on conventional and self-measured blood pressure and cardiovascular risk factors was available; (2) the study was reported as an original research study in a peer-reviewed publication; (3) the study was published between 1 January 1980 and 28 February 2012; (4) the study involved a general population sample; and (5) the subsequent follow-up included both fatal and nonfatal outcomes. After eliminating duplicate population cohorts, the two authors (K. A. and T. J. N.) excluded 57 articles because no home blood pressure (HBP) was measured (*n* = 34), HBP was not self-measured (*n* = 4), the study included patients instead of a population sample (*n* = 7), or cardiovascular outcome was not collected (*n* = 12). J. A. S. and K. A. assessed nine studies in detail [10]–[18] and further eliminated three population cohorts, because no outcome data had been collected [10], only fatal outcomes had been recorded [14], or individual-participant data were unavailable [12].

### Study Population

At the time of writing this report, IDHOCO included six eligible population cohorts [11],[13],[15]–[18] and 8,486 participants (Table 2). We discarded one cohort because data on cause-specific mortality were still being collected [18]. Of the remaining 6,753 participants, we excluded 1,745 because they were on antihypertensive drug treatment initiated before enrollment based on CBP measurement (*n* = 1,465), because fewer than two measurements of their CBP or HBP were on record (*n* = 270), or because information on current smoking or cardiovascular disease history was unavailable (*n* = 10). Thus, the number of participants analyzed totaled 5,008. Analyzed participants were 1,605 Finns representing a nationwide population sample (the Finn-Home study) [17]; 568 inhabitants of Didima, Greece [15]; 2,010 residents from Ohasama, Japan [11]; 469 inhabitants of the Tsurugaya district in Sendai, Japan [16]; and 356 participants living in Montevideo, Uruguay [13].

**Table 2:** Population sampling methods in IDHOCO cohorts.

Catchment Area [Reference]	Sampling Frame	Starting Point	Participation Rate	Recruitment Period	Number in IDHOCO Database	Number Analyzed	Length of Follow-Up (Years)^a^
Finland (nationwide) [17]	Two-stage cluster sample of people aged 45–74 y	Population register to represent Finnish adults	48%	2000–2001	2,075	1,605	8.2 (7.3–8.3)
Dijon, France [18]	Randomly selected people aged ≥65 y	Electoral rolls list	87%	2006–2008	1,733	—	—
Didima, Argolida of Peloponnesus, Greece [15]	People aged ≥18 y	Local registry	76%	1997	665	568	8.2 (7.0–8.4)
Ohasama, Iwate prefecture, Japan [11]	People aged ≥35 y	Address list	80%	1988–1995	2,777	2,010	11.9 (3.8–16.9)
Tsurugaya, Sendai, Japan [16]	People aged ≥70 y	Address list	43%	2002	836	469	5.5 (2.3–5.6)
Montevideo, Uruguay [13]	Age-stratified random sample	Members of a health insurance medical care institution	34% of participants in the study of ambulatory blood pressure	1996–1998	400	356	8.9 (5.7–10.6)

a Median (5th to 95th percentile interval).

### Data Collection

IDHOCO was constructed and maintained at the Studies Coordinating Centre in Leuven, Belgium, in accordance with Belgian legislation on the protection of privacy [8]. Investigators provided anonymous information on each participant in electronic format. After integration of the information into the overarching database, investigators received detailed summary statistics on their own cohort. This procedure ensured that the common database incorporated unbiased information without conflicts between the originally published reports [11],[13],[15]–[17] and results generated for individual studies as part of the current meta-analysis.

### Blood Pressure Measurement

CBP was measured with a standard mercury sphygmomanometer (Mercuro 300 [17] or Baumomanometer [15]), an automatic auscultatory (Elquest USM-700F [11]), or a validated oscillometric monitor (Omron Form ABI/PWV [16] or Omron HEM-705CP [13]), using the appropriate cuff size, with the participant in the sitting or supine position. The CBP was the average of two consecutive readings obtained at an examination center. Next, we classified the CBP according to the generally accepted thresholds that are available in the European [1] and US [2] guidelines. Optimal blood pressure was a level below 120 mm Hg systolic and below 80 mm Hg diastolic. For normal and high-normal blood pressure and mild hypertension, systolic/diastolic levels encompassed 120–129/80–84 mm Hg, 130–139/85–89 mm Hg, and 140–159/90–99 mm Hg, respectively. Severe hypertension was a level of 160 mm Hg systolic or 100 mm Hg diastolic or higher. An additional category, prehypertension, combines participants with normal and high-normal blood pressure (120–139 mm Hg systolic or 80–89 mm Hg diastolic). When the systolic and diastolic blood pressures were in different categories, we assigned the participant to the higher category.

All participants measured their blood pressure at home after 2–5 min of rest in the sitting position with a validated oscillometric device (Omron HEM-722C [17], Omron HEM-705CP [15], Omron HEM-401C [11], Omron HEM-747 [16], or SpaceLabs 90207 [13]) using the appropriate cuff size. Each participant's HBP was the average of all available readings. Masked hypertension was a CBP of less than 140 mm Hg systolic and 90 mm Hg diastolic in the presence of a HBP of 130 mm Hg systolic or 85 mm Hg diastolic or higher [1],[9]. In sensitivity analyses, we also used 135 mm Hg as the systolic threshold for HBP to define masked hypertension [1],[2].

### Other Measurements

Via questionnaires, we obtained information on each participant's medical history and smoking habits. Body mass index was body weight in kilograms divided by height in meters squared. Biochemical measurements included serum cholesterol and blood glucose. Information on serum total cholesterol level was not available for the Didima population and was, as in previous publications [8],[9], extrapolated from data provided by the ATTICA study investigators by sex and 10-y age strata. The ATTICA study population was a large population cohort examined in the same time period and in the same geographical area as the Didima cohort [19],[20]. Diabetes mellitus was defined as the use of antidiabetic drugs, a fasting blood glucose concentration of at least 7.0 mmol/l [21], a random blood glucose concentration of at least 11.1 mmol/l [21], a self-reported diagnosis, or diabetes documented in practice or hospital records.

### Ascertainment of Events

We ascertained vital status and incidence of fatal and nonfatal diseases from the appropriate sources in each country, as described in detail in a previous publication [8]. Fatal and nonfatal stroke did not include transient ischemic attacks. Cardiac events comprised fatal and nonfatal myocardial infarction, death because of ischemic heart disease, sudden death, fatal and nonfatal heart failure, surgical and percutaneous coronary revascularization, pacemaker implantation, and other cardiac deaths. The composite cardiovascular end point included cardiovascular mortality, cerebrovascular, and cardiac end points. In all outcome analyses, we considered only the first occurrence per participant in each event category.

### Statistical Analysis

For database management and statistical analysis, we used SAS software, version 9.3 (SAS Institute). We compared means and proportions using the standard normal *z*-test for large samples or ANOVA and the χ^2^ statistic, respectively. Statistical significance was α level less than 0.05 on two-sided tests. We plotted incidence rates by the five categories of CBP, while standardizing by the direct method for sex and age (<40, 40–59, and ≥60 y).

We used Cox proportional hazards models to compute hazard ratios (HRs), while adjusting for cohort as a random effect and for sex, age, body mass index, smoking, total cholesterol, and history of cardiovascular disease and diabetes mellitus as fixed effects. We checked the proportional hazards assumption and the functional forms of the covariables using the Kolmogorov-type supremum test, as implemented in the PROC PHREG procedure of the SAS package. In multivariable-adjusted Cox proportional hazards models, we explored whether, within each category of CBP, HBP analyzed as continuous variable refined risk stratification. We derived HRs that expressed the change in risk associated with increases in the systolic and diastolic HBP of 10 and 5 mm Hg, respectively. In sensitivity analyses, we computed the differences in the HRs between subgroups by introducing the appropriate interaction term in the Cox proportional hazards models. Finally, we computed multivariable-adjusted HRs comparing the risk of masked hypertension in participants with optimal, normal, or high-normal CBP with the risk incurred by participants with optimal CBP without masked hypertension. We tested heterogeneity in the HRs among the three subgroups with masked hypertension by testing an ordinal variable coding for these subgroups among participants with masked hypertension.

## Results

### Baseline Characteristics

Of 5,008 participants, 2,834 (56.6%) were female, 2,115 (42.2%) were 60 y or older, 1,148 (22.9%) were current smokers, 317 (6.3%) had diabetes mellitus, and 327 (6.5%) had a history of cardiovascular disease. Age averaged 57.1 y (standard deviation [SD], 13.6). Across all participants, CBP averaged 130.9 (SD, 19.7) mm Hg systolic and 77.9 (SD, 11.5) mm Hg diastolic. The corresponding means for HBP were 123.9 (SD, 17.2) mm Hg and 74.9 (SD, 9.8) mm Hg. The mean self-measured HBP was therefore 7.0 mm Hg (95% CI, 6.5–7.4; *p*<0.0001) and 3.0 mm Hg (95% CI, 2.8–3.3; *p*<0.0001) lower than mean CBP. Table 3 lists the baseline characteristics of the 5,008 participants by CBP category. All of the ANOVA and χ^2^ statistic *p*-values for differences across the five categories were significant (*p*≤0.015) except for the prevalence of smoking (*p* = 0.083).

**Table 3:** Participants characteristics according to conventional blood pressure categories.

Characteristic	Optimal (*n* = 1,337)	Normal (*n* = 1,015)	High-Normal (*n* = 1,038)	Mild Hypertension (*n* = 1,126)	Severe Hypertension (*n* = 492)
**Number (percent) with characteristic**					
Women	900 (67.3)	570 (56.2)‡	538 (51.8)*	573 (50.9)	253 (51.4)
Current smoking	333 (24.9)	238 (23.5)	243 (23.4)	236 (21.0)	98 (19.9)
Diabetes mellitus	60 (4.5)	64 (6.3)	74 (7.1)	79 (7.0)	40 (8.1)
Previous cardiovascular diseases	70 (5.2)	55 (5.4)	68 (6.6)	97 (8.6)	37 (7.5)
White (race)	724 (54.2)	457 (45.0)‡	488 (47.0)	589 (52.3)*	271 (55.1)
**Mean (SD) of characteristic**					
Age (years)	50.9 (14.1)	56.1 (12.8)‡	58.5 (12.5)‡	61.0 (12.1)‡	64.1 (11.6)‡
Body mass index (kg/m^2^)	23.9 (3.6)	24.7 (3.8)‡	25.4 (4.0)†	26.1 (4.3)‡	26.6 (4.6)*
Total cholesterol (mmol/l)	5.19 (0.95)	5.38 (1.00)‡	5.43 (1.06)	5.62 (1.12)‡	5.87 (1.22)‡
Conventional systolic pressure (mm Hg)	109.0 (7.6)	123.3 (4.5)‡	132.5 (5.5)‡	145.6 (7.6)‡	168.6 (14.7)‡
Conventional diastolic pressure (mm Hg)	67.6 (6.8)	74.3 (6.6)‡	78.9 (7.8)‡	85.3 (8.3)‡	94.8 (10.9)‡
Home systolic pressure (mm Hg)	110.3 (10.9)	119.3 (12.1)‡	124.4 (12.4)‡	133.8 (14.5)‡	146.5 (17.7)‡
Home diastolic pressure (mm Hg)	68.2 (7.6)	73.1 (8.0)‡	75.4 (8.0)‡	79.7 (8.7)‡	84.8 (10.1)‡

White (race) included Finns, Greeks and Uruguayans. Systolic/diastolic thresholds for CBP were as follows: optimal, <120/<80 mm Hg; normal, 120–129/80–84 mm Hg; high-normal, 130–139/85–89 mm Hg; mild hypertension, 140–159/90–99 mm Hg; and severe hypertension, ≥160/≥100 mm Hg. When the systolic and diastolic blood pressures were in different categories, the participant was assigned to the higher category. All of the ANOVA and χ^2^ statistic *p*-values for differences across the five categories were significant (*p*≤0.015) except for the prevalence of smoking (*p* = 0.083). Significance of the difference with the adjacent lower category of CBP: *p<0.05; †*p*<0.001; and ‡*p*<0.0001.

### Incidence of Events

Median follow-up was 8.3 y (5th to 95th percentile interval, 4.7 to 16.8) and ranged by study from 5.5 y (2.3 to 5.6) in Tsurugaya to 11.9 y (3.8 to 16.9) in Ohasama (Table 2). During 46,593 person-years of follow-up, 522 participants died (11.2 per 1,000 person-years), and 414 experienced a fatal or nonfatal cardiovascular event (9.1 per 1,000 person-years). Considering cause-specific first cardiovascular events, the incidence of stroke and cardiac events amounted to 225 (4.9 per 1,000 person-years) and 194 (4.2 per 1,000 person-years), respectively.

### Risk Associated with Increasing Categories of Conventional Blood Pressure

**Figure 2:**
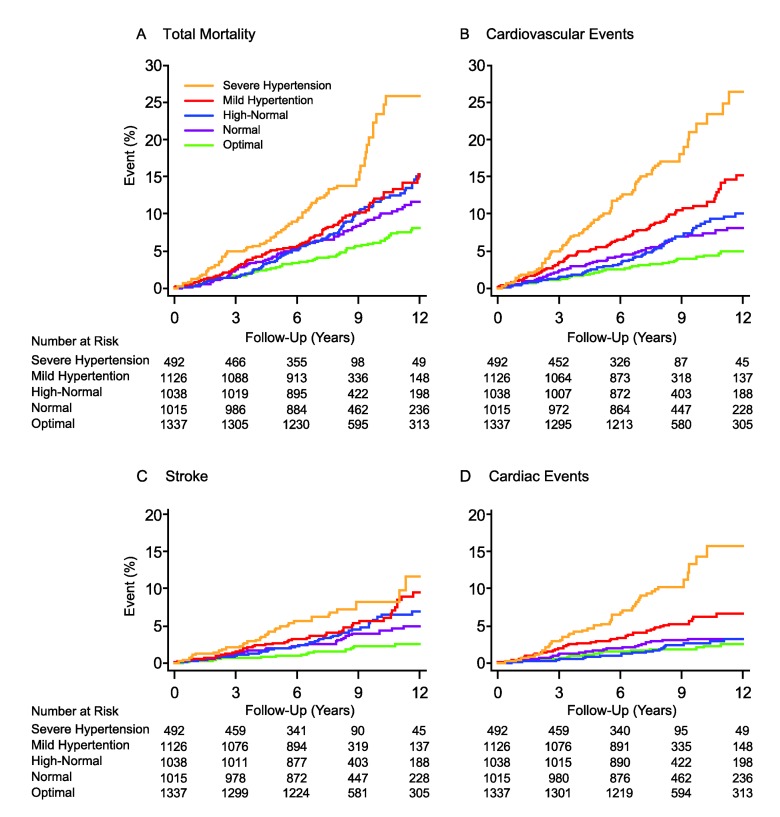
Kaplan-Meier survival function estimates by five categories of conventional blood pressure in 5,008 participants. (A) indicates risk for total mortality, and (B–D) indicate risks for cardiovascular events, stroke, and cardiac events, respectively. CBP categories were optimal (<120/<80 mm Hg), normal (120–129/80–84 mm Hg), high-normal (130–139/85–89 mm Hg), mild hypertension (140–159/90–99 mm Hg), and severe hypertension (≥160/≥100 mm Hg). When the systolic and diastolic blood pressures were in different categories, the participant was assigned to the higher category. The significance of the log-rank test for difference across the five categories was significant (*p*<0.0001) for all of the end points.

**Figure 3:**
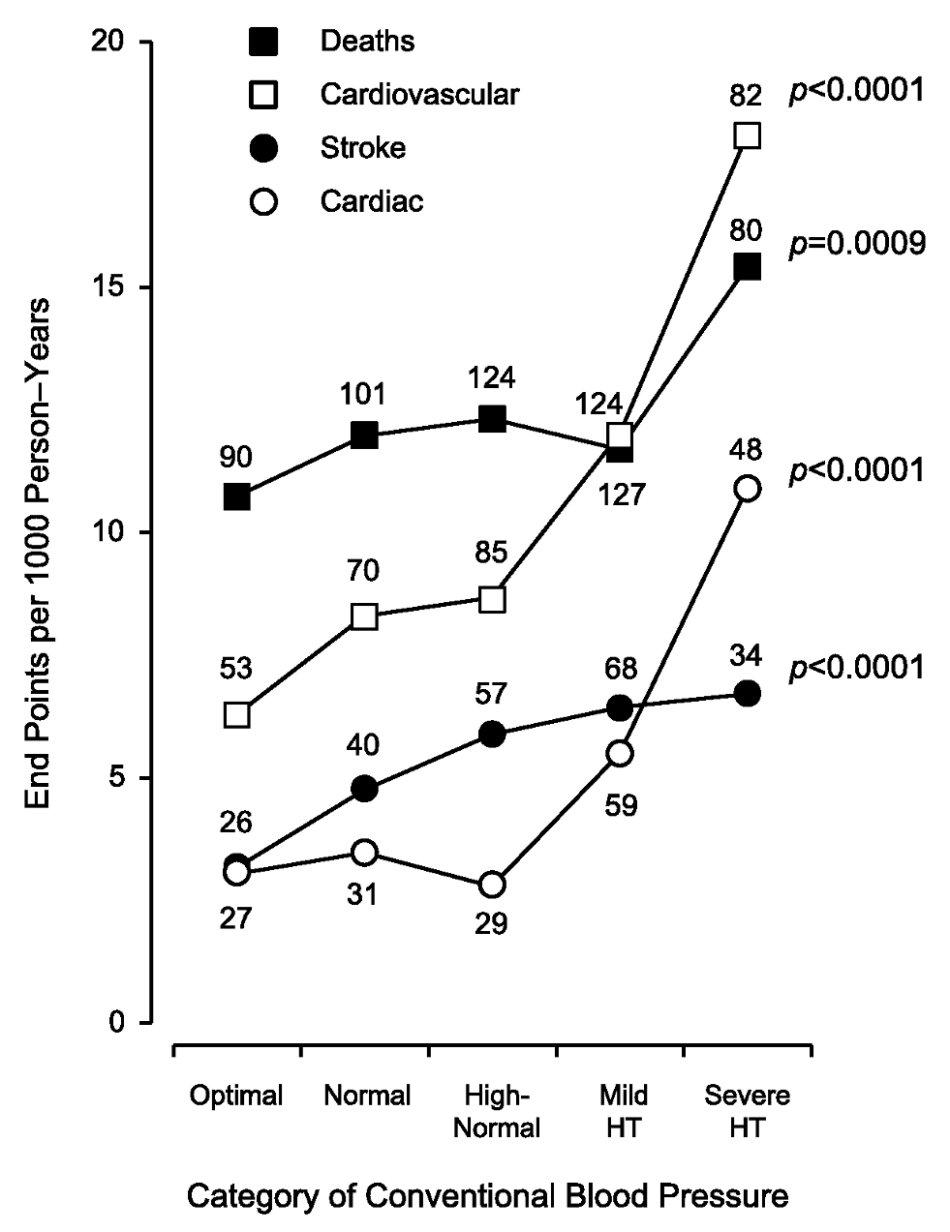
Incidence rates in 5,008 participants by increasing categories of conventional blood pressure. Rates (given as end points per 1,000 person-years) were standardized for sex and age by the direct method. CBP categories were optimal (<120/<80 mm Hg), normal (120–129/80–84 mm Hg), high-normal (130–139/85–89 mm Hg), mild hypertension (140–159/90–99 mm Hg), and severe hypertension (≥160/≥100 mm Hg). When the systolic and diastolic blood pressures were in different categories, the participant was assigned to the higher category. The number of end points contributing to the rates is presented. The *p*-values refer to the significance for linear trend across the five categories of CBP. HT, hypertension.

Figure 2 displays the Kaplan-Meier survival function estimates. The log-rank test for difference across the categories of CBP was highly significant for all of the end points under study (*p*<0.0001). Similarly, incidence rates standardized by the direct method for sex and age (<40, 40–59, and ≥60 y) increased across the categories of CBP (Figure 3; *p*≤0.0009). The multivariable-adjusted HRs, expressing the risk compared with optimal blood pressure (Table 4), increased with higher categories of CBP for total mortality (*p* = 0.011) as well as for fatal combined with nonfatal outcomes (*p*≤0.0004).

**Table 4:** Risks associated with increasing categories of conventional blood pressure.

End Point	Statistic	CBP Category					*p*-Value
		Optimal	Normal	High-Normal	Mild Hypertension	Severe Hypertension	
Total mortality	Number (percent)	90 (6.7)	101 (10.0)	124 (12.0)	127 (11.3)	80 (16.3)	
	HR (95% CI)	1.00	1.15 (0.86–1.53)	1.16 (0.88–1.53)	1.13 (0.86–1.49)	1.71 (1.25–2.33)‡	0.011
Cardiovascular events	Number (percent)	53 (4.0)	70 (6.9)	85 (8.2)	124 (11.0)	82 (16.7)	
	HR (95% CI)	1.00	1.33 (0.93–1.90)	1.33 (0.94–1.89)	1.76 (1.26–2.45)†	2.59 (1.81–3.73)‡	<0.0001
Stroke	Number (percent)	26 (1.9)	40 (3.9)	57 (5.5)	68 (6.0)	34 (6.9)	
	HR (95% CI)	1.00	1.56 (0.95–2.57)	1.89 (1.18–3.02)*	2.24 (1.41–3.56)†	2.72 (1.60–4.62)†	<0.0001
Cardiac events	Number (percent)	27 (2.0)	31 (3.1)	29 (2.8)	59 (5.2)	48 (9.8)	
	HR (95% CI)	1.00	1.16 (0.69–1.96)	0.86 (0.51–1.47)	1.42 (0.88–2.28)	2.40 (1.46–3.96)†	0.0004

Number and HR indicate the number of end points (percentage rate) and HR (95% confidence interval), respectively. Systolic/diastolic thresholds for CBP were as follows: optimal, <120/<80 mm Hg; normal, 120–129/80–84 mm Hg; high-normal, 130–139/85–89 mm Hg; mild hypertension, 140–159/90–99 mm Hg; and severe hypertension, ≥160/≥100 mm Hg. When the systolic and diastolic blood pressures were in different categories, the participant was assigned to the higher category. HRs express the risk compared with optimal blood pressure (reference). HRs were adjusted for cohort as a random effect and for sex, age, body mass index, smoking, total cholesterol, diabetes mellitus, and history of cardiovascular disease as fixed effects. The *p*-value refers to linear trend across the blood pressure categories. Significance of the HRs: *p<0.01; †*p*<0.001; and ‡*p*<0.0001.

### Risk Associated with Home Blood Pressure by Category of Conventional Blood Pressure

Among participants with an optimal CBP, the multivariable-adjusted HRs associated with a 10-mm Hg increment in home systolic blood pressure were 1.21 (95% CI, 1.00–1.46) and 1.28 (95% CI, 1.01–1.62) for total mortality and cardiovascular events. The corresponding HRs among participants with normal CBP were 1.18 (95% CI, 0.99–1.40) and 1.22 (95% CI, 1.00–1.49), respectively. The home systolic blood pressure also predicted the composite cardiovascular end point and stroke (Table 5) in participants with high-normal blood pressure, prehypertension, and mild hypertension. For these categories, the HRs for the composite cardiovascular end point were 1.24 (95% CI, 1.03–1.49), 1.24 (95% CI, 1.09–1.41), and 1.20 (95% CI, 1.06–1.37), respectively, and for stroke were 1.33 (95% CI, 1.07–1.65), 1.27 (95% CI, 1.08–1.50), and 1.30 (95% CI, 1.09–1.56), respectively. However, in participants with severe hypertension, the HBP did not significantly add to the prediction of any end point under study (*p*≥0.20). The home diastolic blood pressure was a weak and inconsistent predictor across the categories of CBP (Table 6).

**Table 5:** Standardized hazard ratios associated with systolic home blood pressure by category of conventional blood pressure.

Category of CBP	HR (95% CI)			
	Total Mortality	Cardiovascular Events	Stroke	Cardiac Events
Optimal	1.21 (1.00–1.46)*	1.28 (1.01–1.62)*	1.26 (0.88–1.79)	1.25 (0.90–1.72)
Normal	1.18 (0.99–1.40)	1.22 (1.00–1.49)*	1.16 (0.89–1.53)	1.29 (0.95–1.75)
High-normal	1.01 (0.86–1.18)	1.24 (1.03–1.49)*	1.33 (1.07–1.65)†	1.03 (0.74–1.43)
Prehypertension	1.08 (0.96–1.21)	1.24 (1.09–1.41)†	1.27 (1.08–1.50)†	1.15 (0.93–1.44)
Mild hypertension	1.04 (0.92–1.18)	1.20 (1.06–1.37)†	1.30 (1.09–1.56)†	1.13 (0.94–1.36)
Severe hypertension	0.95 (0.83–1.09)	0.95 (0.83–1.08)	1.00 (0.82–1.23)	0.89 (0.74–1.06)
Hypertension	1.04 (0.96–1.14)	1.12 (1.02–1.22)*	1.19 (1.05–1.35)†	1.05 (0.93–1.20)

Systolic/diastolic thresholds for CBP were as follows: optimal, <120/<80 mm Hg; normal, 120–129/80–84 mm Hg; high-normal, 130–139/85–89 mm Hg; mild hypertension, 140–159/90–99 mm Hg; and severe hypertension, ≥160/≥100 mm Hg. When the systolic and diastolic blood pressures were in different categories, the participant was assigned to the higher category. The category prehypertension includes participants with normal and high-normal blood pressure, and the category hypertension includes participants with mild and severe hypertension. The number of people at risk and the number of events are given in Tables 3 and 4, respectively. HRs reflect the risk associated with a 10-mm Hg increase in home systolic pressure. HRs were adjusted for cohort as a random effect and for sex, age, body mass index, smoking, total cholesterol, diabetes mellitus, and history of cardiovascular disease as fixed effects. Significance of the HRs: *p<0.05 and †*p*<0.01.

**Table 6:** Standardized hazard ratios associated with diastolic home blood pressure by category of conventional blood pressure.

Category of CBP	HR (95% CI)			
	Total Mortality	Cardiovascular Events	Stroke	Cardiac Events
Optimal	1.04 (0.91–1.19)	1.07 (0.89–1.29)	0.95 (0.72–1.24)	1.19 (0.92–1.55)
Normal	1.01 (0.89–1.14)	1.03 (0.88–1.20)	1.02 (0.83–1.26)	1.05 (0.82–1.35)
High-normal	0.95 (0.84–1.06)	1.09 (0.95–1.26)	1.11 (0.94–1.31)	1.00 (0.77–1.31)
Prehypertension	0.98 (0.90–1.07)	1.07 (0.97–1.19)	1.08 (0.95–1.23)	1.03 (0.86–1.24)
Mild hypertension	1.02 (0.92–1.13)	1.21 (1.09–1.34)‡	1.26 (1.10–1.44)‡	1.13 (0.96–1.33)
Severe hypertension	0.90 (0.80–1.02)	0.91 (0.81–1.04)	1.04 (0.87–1.25)	0.81 (0.68–0.96)*
Hypertension	1.01 (0.93–1.08)	1.11 (1.03–1.20)†	1.20 (1.08–1.34)‡	1.00 (0.89–1.19)

Systolic/diastolic thresholds for CBP were as follows: optimal, <120/<80 mm Hg; normal, 120–129/80–84 mm Hg; high-normal, 130–139/85–89 mm Hg; mild hypertension, 140–159/90–99 mm Hg; and severe hypertension, ≥160/≥100 mm Hg. When the systolic and diastolic blood pressures were in different categories, the participant was assigned to the higher category. The category prehypertension includes participants with normal and high-normal blood pressure, and the category hypertension includes participants with mild and severe hypertension. The number of people at risk and the number of events are given in Tables 3 and 4, respectively. HRs reflect the risk associated with a 5-mm Hg increase in home diastolic pressure. HRs were adjusted for cohort as a random effect and for sex, age, body mass index, smoking, total cholesterol, diabetes mellitus, and history of cardiovascular disease as fixed effects. Significance of the HRs: *p<0.05; †*p*<0.01; and ‡*p*<0.001.

Excluding one cohort at a time or stratifying participants by sex, ethnicity, or age (<60 versus ≥60 y) confirmed the main analyses of home systolic blood pressure, as reported in Tables 7 and 8.

**Table 7:** Sensitivity analysis for total mortality and cardiovascular events with one cohort excluded.

End Point	Finn-Home Excluded		Didima Excluded		Ohasama Excluded		Tsurugaya Excluded		Montevideo Excluded	
	E/R	HR (95% CI)	E/R	HR (95% CI)	E/R	HR (95% CI)	E/R	HR (95% CI)	E/R	HR (95% CI)
**Total mortality**										
Optimal	76/1,043	1.13 (0.91–1.40)	83/1,098	1.26 (1.04–1.53)*	30/779	1.08 (0.79–1.46)	83/1,282	1.27 (1.04–1.55)*	88/1,146	1.20 (0.99–1.46)
Normal	83/724	1.15 (0.95–1.39)	96/922	1.19 (1.00–1.42)*	31/532	1.43 (1.08–1.91)*	93/940	1.14 (0.94–1.38)	101/942	1.18 (0.99–1.40)
High-normal	109/694	0.99 (0.84–1.18)	114/940	1.00 (0.84–1.18)	34/576	0.93 (0.70–1.24)	116/950	1.04 (0.88–1.23)	123/992	1.01 (0.86–1.19)
Mild hypertension	100/669	1.05 (0.91–1.21)	113/1,027	1.09 (0.96–1.23)	52/742	0.92 (0.75–1.14)	116/973	1.07 (0.94–1.21)	127/1,093	1.04 (0.92–1.18)
Severe hypertension	62/273	0.87 (0.75–1.01)	74/453	0.93 (0.80–1.07)	34/369	1.08 (0.87–1.33)	72/394	0.96 (0.83–1.10)	78/479	0.96 (0.83–1.10)
**Cardiovascular events**										
Optimal	37/1,043	1.21 (0.89–1.65)	49/1,098	1.36 (1.07–1.72)*	25/779	1.49 (1.08–2.06)*	50/1,282	1.13 (0.87–1.45)	51/1,146	1.31 (1.03–1.67)*
Normal	51/724	1.24 (0.98–1.57)	65/922	1.27 (1.03–1.56)*	32/532	1.10 (0.81–1.49)	64/940	1.22 (0.97–1.54)	68/942	1.26 (1.03–1.54)*
High-normal	65/694	1.33 (1.08–1.63)†	80/940	1.23 (1.02–1.48)*	30/576	1.08 (0.79–1.48)	83/950	1.22 (1.01–1.47)*	82/992	1.27 (1.05–1.52)*
Mild hypertension	84/669	1.26 (1.07–1.48)†	111/1,027	1.24 (1.09–1.42)†	67/742	1.08 (0.91–1.29)	112/973	1.22 (1.07–1.40)†	122/1,093	1.20 (1.05–1.37)†
Severe hypertension	48/273	0.87 (0.74–1.03)	77/453	0.94 (0.82–1.08)	52/369	0.95 (0.80–1.14)	73/394	0.99 (0.86–1.15)	78/479	0.94 (0.82–1.09)

E/R indicates the number of cardiovascular events/participants at risk. Systolic/diastolic thresholds for CBP were as follows: optimal, <120/<80 mm Hg; normal, 120–129/80–84 mm Hg; high-normal, 130–139/85–89 mm Hg; mild hypertension, 140–159/90–99 mm Hg; and severe hypertension, ≥160/≥100 mm Hg. When the systolic and diastolic blood pressures were in different categories, the participant was assigned to the higher category. HRs reflect the risk for a 10-mm Hg increase in home systolic pressure and were adjusted for cohort as a random effect and for sex, age, body mass index, smoking, total cholesterol, diabetes mellitus, and history of cardiovascular disease as fixed effects. Significance of the HRs: *p<0.05 and †*p*<0.01.

**Table 8:** Sensitivity analysis for total mortality and cardiovascular events according to anthropometric characteristics and cardiovascular risk factors.

End Point	Subgroup	Statistic	CBP				
			Optimal	Normal	High-Normal	Mild Hypertension	Severe Hypertension
**Total mortality**	**Women**	E/R	37/900	40/570	54/538	43/573	30/253
		HR (95% CI)	1.19 (0.88–1.62)	1.24 (0.95–1.61)	0.96 (0.74–1.25)	1.04 (0.83–1.31)	1.00 (0.79–1.27)
	**Men**	E/R	53/437	61/445	70/500	84/553	50/239
		HR (95% CI)	1.24 (0.97–1.58)	1.15 (0.91–1.45)	1.06 (0.86–1.30)	0.99 (0.84–1.16)	0.97 (0.82–1.16)
	**<60 y**	E/R	28/985	26/636	30/559	25/538	10/175
		HR (95% CI)	1.27 (0.85–1.88)	1.52 (1.06–2.20)*	1.26 (0.91–1.77)	1.06 (0.74–1.50)	2.02 (1.20–3.40)†
	**≥60 y**	E/R	62/352	75/379	94/479	102/588	70/317
		HR (95% CI)	1.20 (0.96–1.50)	1.14 (0.94–1.39)	0.95 (0.79–1.14)	1.05 (0.92–1.20)	0.90 (0.78–1.04)
	**Japanese**	E/R	67/613	78/558	98/550	86/537	54/221
		HR (95% CI)	1.20 (0.96–1.51)	1.16 (0.96–1.40)	0.99 (0.83–1.19)	1.11 (0.96–1.28)	0.82 (0.68–0.97)*
							§
	**White (race)**	E/R	23/724	23/457	26/488	41/589	26/271
		HR (95% CI)	1.27 (0.86–1.88)	1.38 (0.93–2.04)	1.06 (0.74–1.50)	0.92 (0.72–1.17)	1.17 (0.91–1.49)
							
	**Nonsmokers**	E/R	56/1,004	69/777	85/795	93/890	63/394
		HR (95% CI)	1.18 (0.94–1.49)	1.23 (1.00–1.51)*	0.90 (0.74–1.11)	0.92 (0.79–1.08)	0.91 (0.78–1.05)
	**Smokers**	E/R	34/333	32/238	39/243	34/236	17/98
		HR (95% CI)	1.28 (0.93–1.77)	1.14 (0.83–1.58)	1.33 (1.01–1.74)*	1.23 (0.99–1.53)	1.27 (0.89–1.81)
	**Body mass index <25 kg/m2**	E/R	70/921	75/592	88/546	84/525	55/206
		HR (95% CI)	1.26 (1.01–1.58)*	1.16 (0.96–1.40)	0.98 (0.81–1.19)	1.08 (0.94–1.25)	0.92 (0.78–1.09)
	**Body mass index ≥25 kg/m2**	E/R	20/416	26/423	36/492	43/601	25/286
		HR (95% CI)	1.13 (0.75–1.71)	1.23 (0.81–1.87)	1.12 (0.82–1.54)	0.98 (0.77–1.24)	0.87 (0.67–1.14)
	**Serum cholesterol <5.69 mmol/l**	E/R	77/1,006	80/684	102/675	99/658	59/240
		HR (95% CI)	1.22 (0.99–1.50)	1.24 (1.02–1.49)*	0.99 (0.83–1.19)	1.01 (0.88–1.16)	0.89 (0.76–1.04)
	**Serum cholesterol ≥5.69 mmol/l**	E/R	13/331	21/331	22/363	28/468	21/252
		HR (95% CI)	1.07 (0.64–1.81)	0.80 (0.49–1.29)	1.07 (0.77–1.50)	1.10 (0.84–1.44)	1.24 (0.94–1.63)
**Cardiovascular events**	**Women**	E/R	26/900	26/570	37/538	40/573	28/253
		HR (95% CI)	1.32 (0.96–1.82)	1.30 (0.96–1.77)	1.16 (0.85–1.56)	1.34 (1.06–1.68)*	0.93 (0.74–1.16)
	**Men**	E/R	27/437	44/445	48/500	84/553	54/239
		HR (95% CI)	1.26 (0.88–1.80)	1.17 (0.90–1.53)	1.33 (1.06–1.68)*	1.15 (0.97–1.35)	0.99 (0.84–1.18)
	**<60 y**	E/R	19/985	26/636	24/559	32/538	20/175
		HR (95% CI)	1.71 (1.08–2.71)*	1.44 (0.98–2.11)	1.35 (0.94–1.94)	1.15 (0.85–1.57)	1.18 (0.88–1.59)
	**≥60 y**	E/R	34/352	44/379	61/479	92/588	62/317
		HR (95% CI)	1.18 (0.87–1.61)	1.24 (0.97–1.58)	1.21 (0.98–1.50)	1.21 (1.05–1.39)†	0.91 (0.78–1.05)
	**Japanese**	E/R	31/613	44/558	57/550	69/537	39/221
		HR (95% CI)	1.43 (1.03–1.99)*	1.39 (1.09–1.76)†	1.37 (1.10–1.70)†	1.33 (1.12–1.58)†	0.81 (0.66–0.99)*
							§
	**White (race)**	E/R	22/724	26/457	28/488	55/589	43/271
		HR (95% CI)	1.17 (0.80–1.71)	0.95 (0.64–1.43)	0.99 (0.69–1.40)	1.06 (0.87–1.29)	1.05 (0.86–1.28)
							
	**Nonsmokers**	E/R	40/1004	51/777	55/795	93/890	62/394
		HR (95% CI)	1.44 (1.11–1.88)†	1.13 (0.89–1.44)	1.26 (1.00–1.59)*	1.13 (0.97–1.31)	0.93 (0.80–1.07)
	**Smokers**	E/R	13/333	19/238	30/243	31/236	20/98
		HR (95% CI)	1.02 (0.61–1.69)	1.44 (0.96–2.17)	1.25 (0.91–1.71)	1.38 (1.07–1.79)*	1.03 (0.75–1.40)
	**Body mass index <25 kg/m2**	E/R	35/921	46/592	54/546	64/525	35/206
		HR (95% CI)	1.55 (1.14–2.10)†	1.29 (1.03–1.62)*	1.20 (0.96–1.51)	1.34 (1.13–1.59)‡	0.86 (0.70–1.06)
	**Body mass index ≥25 kg/m2**	E/R	18/416	24/423	31/492	60/601	47/286
		HR (95% CI)	1.02 (0.64–1.61)	0.97 (0.64–1.47)	1.39 (1.01–1.92)*	1.05 (0.85–1.28)	0.94 (0.78–1.14)
	**Serum cholesterol <5.69 mmol/l**	E/R	40/1,006	48/684	62/675	84/658	48/240
		HR (95% CI)	1.46 (1.12–1.91)†	1.37 (1.08–1.74)†	1.43 (1.15–1.78)†	1.19 (1.02–1.40)*	0.95 (0.79–1.13)
	**Serum cholesterol ≥5.69 mmol/l**	E/R	13/331	22/331	23/363	40/468	34/252
		HR (95% CI)	0.95 (0.52–1.71)	0.95 (0.64–1.42)	0.91 (0.62–1.33)	1.24 (0.98–1.55)	1.00 (0.80–1.23)

E/R indicates the number of end points/participants at risk. White (race) included Finns, Greeks, and Uruguayans. Systolic/diastolic thresholds for CBP were as follows: optimal, <120/<80 mm Hg; normal, 120–129/80–84 mm Hg; high-normal, 130–139/85–89 mm Hg; mild hypertension, 140–159/90–99 mm Hg; and severe hypertension, ≥160/≥100 mm Hg. When the systolic and diastolic blood pressures were in different categories, the participant was assigned to the higher category. HRs reflect the risk for a 10-mm Hg increase in home systolic pressure and were adjusted for cohort as a random effect and for sex, age, body mass index, smoking, total cholesterol, diabetes mellitus, and history of cardiovascular disease as fixed effects. Significance of the HRs: *p<0.05; †*p*<0.01; and ‡*p*<0.001.

§indicates a significant difference (*p*≤0.05) in the HRs between corresponding strata.

### Characteristics of Participants with Masked Hypertension

Participants with masked hypertension according to the 130/85-mm Hg threshold, compared with participants with true optimal, normal, or high-normal blood pressure (Table 9), were more likely to be male (51.0% versus 38.7%; *p*<0.0001), to smoke (28.1% versus 23.2%; *p* = 0.012), to have diabetes mellitus (10.5% versus 4.9%; *p*<0.0001) or a history of cardiovascular disease (8.3% versus 5.2%; *p* = 0.0038), and to be older (62.9 versus 53.2 y; *p*<0.0001) and more obese (26.0 versus 24.3 kg/m^2^; *p*<0.0001).

**Table 9:** Characteristics of participants with masked hypertension (home blood pressure ≥130/≥85 mm Hg) compared with participants with true optimal, normal, or high-normal blood pressure (home blood pressure <130/<85 mm Hg).

Characteristic	HBP <130/<85 mm Hg			HBP ≥130/≥85 mm Hg		
	Optimal (*n* = 1,270)	Normal (*n* = 828)	High-Normal (*n* = 723)	Optimal (*n* = 67)	Normal (*n* = 187)	High-Normal (*n* = 315)
**Number (percent) with characteristic**						
Women	859 (67.6)	479 (57.9)§	391 (54.1)	41 (61.2)	91 (48.7)	147 (46.7)
Current smoking	316 (24.9)	189 (22.8)	149 (20.6)	17 (25.4)	49 (26.2)	94 (29.8)
Diabetes mellitus	53 (4.2)	45 (5.4)	40 (5.5)	7 (10.5)	19 (10.2)	34 (10.8)
Previous cardiovascular diseases	62 (4.9)	37 (4.5)	47 (6.5)	8 (12.0)	18 (9.6)	21 (6.7)
**Mean characteristic (SD)**						
Age (years)	50.4 (13.9)	54.5 (12.5)§	56.5 (12.5)†	61.3 (13.5)	63.2 (11.7)	63.0 (11.4)
Body mass index (kg/m^2^)	23.9 (3.6)	24.5 (3.6)‡	25.0 (3.7)*	24.8 (4.1)	25.8 (4.3)	26.3 (4.5)
Total cholesterol (mmol/l)	5.20 (0.94)	5.38 (1.01)§	5.40 (1.04)	5.11 (1.08)	5.34 (0.97)	5.50 (1.09)

Systolic/diastolic thresholds for CBP were as follows: optimal, <120/<80 mm Hg; normal, 120–129/80–84 mm Hg; and high-normal, 130–139/85–89 mm Hg. When the systolic and diastolic blood pressures were in different categories, the participant was assigned to the higher category. Significance of the difference from the adjacent lower category of CBP: *p<0.05; †*p*<0.01; ‡*p*<0.001; and §*p*<0.0001.

Participants with masked hypertension according to the 135/85-mm Hg threshold, compared with participants with true optimal, normal, or high-normal blood pressure (Table 10), were more likely to be male (55.7% versus 38.7%; *p*<0.0001), to smoke (29.6% versus 23.3%; *p* = 0.0053), to have diabetes mellitus (11.6% versus 5.1%; *p*<0.0001) or a history of cardiovascular disease (9.1% versus 5.2%; *p* = 0.0015), and to be older (62.8 versus 53.7 y; *p*<0.0001) and more obese (26.5 versus 24.4 kg/m^2^; *p*<0.0001).

**Table 10:** Characteristics of participants with masked hypertension (home blood pressure ≥135/≥85 mm Hg) compared with participants with true optimal, normal, or high-normal blood pressure (home blood pressure <135/<85 mm Hg).

Characteristic	HBP <135/<85 mm Hg			HBP ≥135/≥85 mm Hg		
	Optimal (*n* = 1,295)	Normal (*n* = 884)	High-Normal (*n* = 805)	Optimal (*n* = 42)	Normal (*n* = 131)	High-Normal (*n* = 233)
**Number (percent) with characteristic**						
Women	874 (67.5)	513 (58.0)‡	441 (54.8)	26 (61.9)	57 (43.5)*	97 (41.6)
Current smoking	321 (24.8)	202 (22.9)	171 (21.2)	12 (28.6)	36 (27.5)	72 (30.9)
Diabetes mellitus	55 (4.3)	50 (5.7)	46 (5.7)	5 (11.9)	14 (10.7)	28 (12.0)
Previous cardiovascular diseases	66 (5.1)	39 (4.4)	51 (6.3)	4 (9.5)	16 (12.2)	17 (7.3)
**Mean characteristic (SD)**						
Age (years)	50.6 (14.0)	55.1 (12.7)‡	57.3 (12.5)†	62.0 (11.2)	63.3 (11.7)	62.6 (11.6)
Body mass index (kg/m^2^)	23.9 (3.6)	24.5 (3.6)†	25.0 (3.7)*	25.4 (4.2)	26.2 (4.6)	26.8 (4.8)
Total cholesterol (mmol/l)	5.20 (0.94)	5.39 (1.00)‡	5.39 (1.05)	5.06 (1.15)	5.29 (0.98)	5.56 (1.08)*

Systolic/diastolic thresholds for CBP were as follows: optimal, <120/<80 mm Hg; normal, 120–129/80–84 mm Hg; and high-normal, 130–139/85–89 mm Hg. When the systolic and diastolic blood pressures were in different categories, the participant was assigned to the higher category. Significance of the difference from the adjacent lower category of CBP: *p<0.05; †*p*<0.001; and ‡*p*<0.0001.

### Risk Conferred by Masked Hypertension

**Figure 4:**
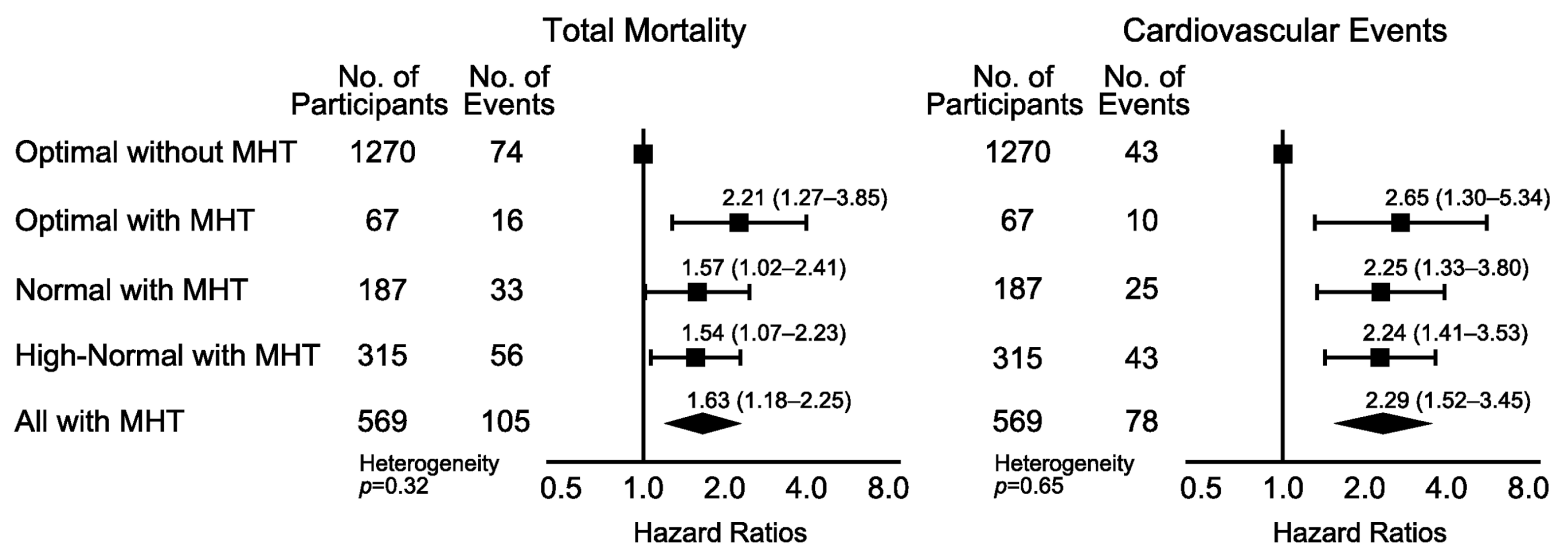
Hazard ratios associated with masked hypertension (≥130/≥85 mm Hg) in participants with optimal, normal, and high-normal conventional blood pressure. Participants with optimal blood pressure without elevated HBP were the reference group. The categories of CBP were optimal (<120/<80 mm Hg), normal (120–129/80–84 mm Hg), and high-normal (130–139/85–89 mm Hg). When the systolic and diastolic blood pressures were in different categories, the participant was assigned to the higher category. Systolic/diastolic thresholds for hypertension on home measurement were ≥130/≥85 mm Hg. The HRs were adjusted for cohort as random effect and for sex, age, body mass index, smoking, total cholesterol, diabetes mellitus, and history of cardiovascular disease as fixed effects. Horizontal lines denote the 95% confidence interval. The diamond represents the pooled estimate in all participants with masked hypertension (MHT). The *p*-value for heterogeneity was derived by testing an ordinal variable in Cox proportional hazards regression coding for the three subgroups among participants with masked hypertension.

Using 130/85 mm Hg as threshold for HBP to define masked hypertension (Figure 4; Table 9), the number of participants with masked hypertension amounted to 67 (5.0%), 187 (18.4%), and 315 (30.3%) among participants with optimal, normal, and high-normal blood pressure, respectively. In these three categories of participants with masked hypertension, with optimal blood pressure without masked hypertension as reference, the multivariable-adjusted HRs for total mortality were 2.21 (95% CI, 1.27–3.85), 1.57 (95% CI, 1.02–2.41), and 1.54 (95% CI, 1.07–2.23), respectively. The corresponding HRs for the composite cardiovascular end point were 2.65 (95% CI, 1.30–5.34), 2.25 (95% CI, 1.33–3.80), and 2.24 (95% CI, 1.41–3.53), respectively.

**Figure 5:**
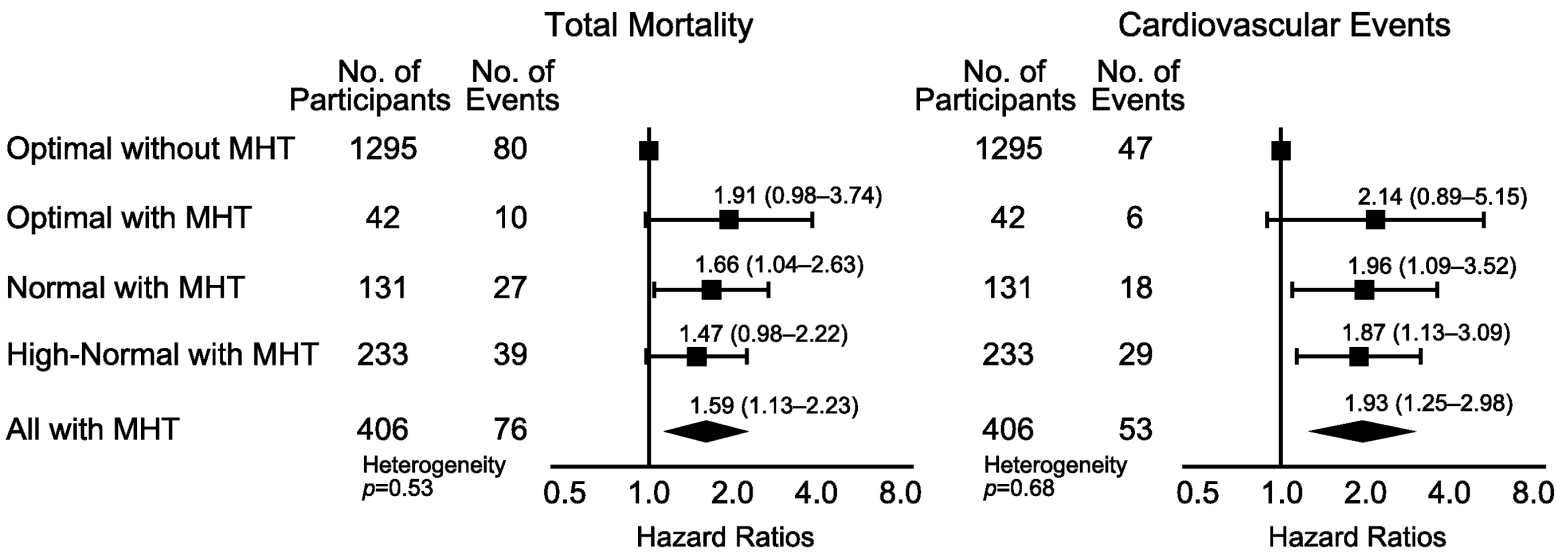
Hazard ratios associated with masked hypertension (≥135/≥85 mm Hg) in participants with optimal, normal, and high-normal conventional blood pressure. Participants with optimal blood pressure without elevated HBP were the reference group. CBP categories were optimal (<120/<80 mm Hg), normal (120–129/80–84 mm Hg), and high-normal (130–139/85–89 mm Hg). When the systolic and diastolic blood pressures were in different categories, the participant was assigned to the higher category. Systolic/diastolic thresholds for hypertension on home measurement were ≥135/≥85 mm Hg. The HRs were adjusted for cohort as a random effect and for sex, age, body mass index, smoking, total cholesterol, diabetes mellitus, and history of cardiovascular disease as fixed effects. Horizontal lines denote the 95% confidence interval. The diamond represents the pooled estimate in all participants with masked hypertension (MHT). The *p*-value for heterogeneity was derived by testing an ordinal variable in Cox proportional hazards regression coding for the three subgroups among participants with masked hypertension.

Using 135/85 mm Hg as the HBP threshold to define masked hypertension (Figure 5; Table 10), the number of participants with masked hypertension amounted to 42 (3.1%), 131 (12.9%), and 233 (22.4%) among participants with optimal, normal, and high-normal blood pressure, respectively. In these three categories of participants with masked hypertension, with optimal blood pressure without masked hypertension as reference, the multivariable-adjusted HRs for total mortality were 1.91 (95% CI, 0.98–3.74), 1.66 (95% CI, 1.04–2.63), and 1.47 (95% CI, 0.98–2.22), respectively. The corresponding HRs for the composite cardiovascular end point were 2.14 (95% CI, 0.89–5.15), 1.96 (95% CI, 1.09–3.52), and 1.87 (95% CI, 1.13–3.09), respectively.

## Discussion

Current guidelines for the diagnosis and management of hypertension [1],[2] stratify risk and treatment decisions based on defined categories of CBP (Table 1), as measured in a medical environment. Self-measured HBP or ambulatory blood pressure is a more accurate prognosticator than CBP. Expert committees therefore recommend the use of out-of-the-office blood pressure measurement to confirm the diagnosis of hypertension and assess treatment effects [1],[2]. The key finding of our current study is that HBP substantially refines risk stratification at levels of CBP that are presumably associated with no or only mildly elevated risk. In contrast, in severe hypertension, self-measured HBP did not improve the prediction of death or cardiovascular complications.

To the best of our knowledge, only one previous prospective study addressed risk stratification by out-of-the-office blood pressure measurement in prehypertensive patients, but applied ambulatory monitoring instead of self-measurement. Pierdomenico and coworkers [22] followed the incidence of cardiovascular events in prehypertensive participants with (*n* = 120) or without (*n* = 471) masked hypertension. The participants were hospital staff, patients referred for reasons other than cardiovascular disease or hypertension, and volunteers. During 6.6 y of follow-up, 29 fatal and nonfatal cardiovascular events occurred. In prehypertensive patients without and with masked hypertension, the event rates per 100 patient-years were 0.57 and 1.51, respectively. With adjustments applied for covariables, including CBP, cardiovascular risk was significantly higher in masked hypertensive patients than in true prehypertension (masked versus true prehypertension without elevated ambulatory blood pressure, relative risk 2.65; 95% CI, 1.18–5.98; *p* = 0.018).

According to the European Society of Hypertension guidelines [1], masked hypertension is a clinic blood pressure below 140 mm Hg systolic and 90 mm Hg diastolic in the presence of a daytime or self-measured blood pressure of at least 130 mm Hg or 135 mm Hg systolic or 85 mm Hg diastolic. The prevalence of masked hypertension in our current study was 8.4%, which is of the same order of magnitude as in other individual-participant meta-analyses of population samples [23],[24]. If one accounts for differences in the technique of out-of-the-office blood pressure measurement, treatment status, and applied thresholds, masked hypertension carries a risk that approaches that of sustained hypertension [23],[24]. In keeping with our current findings (Tables 9 and 10), the Finn-Home study investigators demonstrated that high-normal systolic and diastolic office blood pressure, older age, greater body mass index, current smoking, and diabetes were independent determinants of masked hypertension [25]. Similarly, Franklin and coworkers [24] reported that among people not being treated for hypertension, the prevalence of masked hypertension, using a daytime ambulatory threshold of 135/85 mm Hg, was higher in diabetic than nondiabetic participants (18.1% versus 13.8%; *p* = 0.032).

Our current findings have important implications for clinical practice. The relation between cardiovascular complications and blood pressure is continuous at least down to a CBP level of 115 mm Hg systolic or 75 mm Hg diastolic [26]. The continuous nature of the relation with blood pressure holds true not only in hypertensive patients, but in normotensive people as well, so that, for instance, of all strokes, three-fourths occur in individuals with normal CBP and only one-fourth in patients with hypertension as determined by CBP [26]. In our current analysis, we demonstrated that HBP monitoring substantially refines risk stratification in normotensive people not being treated for hypertension with optimal, normal, or high-normal CBP, in particular in the presence of masked hypertension. Consequently, we suggest that in individuals at risk for masked hypertension, HBP monitoring should be included in the strategy of primary prevention of cardiovascular complications. Risk factors that identify people who would qualify for HBP monitoring are above optimal levels of CBP, older age, obesity, smoking, and diabetes mellitus [24],[25]. HBP monitoring should also be carried out in people with a normal CBP but with unexplained signs of hypertensive target organ damage.

Using HBP measurement to screen for masked hypertension is probably cost-effective. Fukunaga and coworkers studied the cost-effectiveness of HBP measurement from the perspective of the Japanese health care system, using simulations based on the Ohasama population study [27]. Depending on the model applied, estimates of the cost savings produced by applying HBP measurement ranged from US$674,000 to US$2.51 million per 5,000 person-years. Two trials of adjusting treatment based on out-of-the-office blood pressure measurement [28],[29] also reported cost savings compared to conventional sphygmomanometry, by using either ambulatory monitoring [28] or self-measurement [29] of blood pressure.

Our individual-participant meta-analysis is clearly an advance over previous publications in the research field of risk stratification based on blood pressure. To our knowledge, our study is the first to assess the risk associated with self-measured HBP across increasing categories of CBP. It raises the issue that the economic analysis of HBP monitoring should be based on cost-savings not only via the avoidance of unnecessary treatment [27]–[29], but foremost via the addition of quality and years to life in those at high cardiovascular risk even though CBP is normal. Other strong points are the relatively large sample size representing populations from Europe, Asia, and South America, and the removal of participants being treated for hypertension from the analysis. Our meta-analysis is also an advance over the contributing individual studies, because none of them had enough power to address the research question that we set out to answer or to run sensitivity analyses stratified by anthropometric characteristics or cardiovascular risk factors. Furthermore, meta-analyses based on individual participant data, compared with meta-analyses of summary statistics, have unique advantages, including the possibility of computing survival curves and the ability to check whether the Cox proportional hazards assumption is fulfilled [30],[31]. Finally, we analyzed both fatal and nonfatal outcomes. The introduction of stroke units to hospitals and the increasing availability of invasive coronary care and thrombolysis over the past decades has reduced the case fatality rate of most cardiovascular complications of hypertension. Not accounting for nonfatal events therefore seriously limits generalizability [32].

On the other hand, our study also has some potential limitations. First, we did not determine the reproducibility of masked hypertension in the context of our current study. However, Viera and colleagues reported prevalence rates of masked hypertension among patients not being treated with hypertension but with borderline elevated CBP to be 54% and 53% on first and repeat assessment, with an agreement of 73% [33]. Among patients who underwent repeated ambulatory monitoring for a medical indication, Ben-Dov and coworkers reported an agreement of 72% in the classification of masked hypertension [34]. Second, CBP values in our study were the average of only two readings obtained at a single examination, which can lead to an overestimation of CBP because of the white-coat effect. However, overestimation of CBP would weaken rather than strengthen our current findings. Moreover, our findings were consistent using a tight (130 mm Hg) and less stringent (135 mm Hg) systolic threshold to define masked hypertension. Third, IDHOCO has no information on treatment status during follow-up. However, antihypertensive treatment instituted during follow-up likely would have been instituted according to current guidelines and based on CBP. Even if treatment had been started during follow-up based on HBP, if anything this would have weakened associations between outcomes and HBP (including masked hypertension). Fourth, few data were available from low- and middle-income countries. Finally, methods of blood pressure measurement and ascertainment of events were not identical among cohorts. However, we accounted for cohort as a random effect in the Cox proportional hazards models, and our findings remained consistent when we excluded one cohort at a time.

In conclusion, HBP monitoring substantially refines risk stratification in people with levels of CBP assumed to carry no or only mildly elevated risk, in particular in the presence of masked hypertension. Properly designed randomized clinical trials are required to determine whether identification and treatment of masked hypertension versus the current standard of care, i.e., not to perform HBP measurement and not to treat people with normal blood pressure on conventional measurement, leads to a reduction of cardiovascular complications and is cost-effective. Such trials might be mounted worldwide in developed and developing countries, including remote areas and even in low-resource settings [35].

## Supporting Information

Text S1 **PRISMA statement.**
(DOC)

## Abbreviations

CBP: conventional blood pressure
HBP: home blood pressure
HR: hazard ratio
IDHOCO: International Database of Home Blood Pressure in Relation to Cardiovascular Outcome
SD: standard deviation
